# Supplementary material for: Cloud BioLinux: pre-configured and on-demand bioinformatics computing for the genomics community
Source: BMC Bioinformatics. 2012 Mar 19;13:42. doi: 10.1186/1471-2105-13-42 (PMC3372431; doi:10.1186/1471-2105-13-42)
Supplement: Additional file 1 — Supplementary 1 Cloud BioLinux software documentation in the form of a mini, self-contained website. Users need to download and uncompress the .zip file, and open through a web browser the "index.html" file available on the main directory. (ZIP 1823 kb). [file 1471-2105-13-42-S1.ZIP › Cloud-BioLinux-Package-Documentation/docs/netblast.html]

Bio-Linux Software Documentation Pages

Back to search form

## netblast

|  |  |
| --- | --- |
| Name | netblast |
| Description | Please consider using **blast+** instead of **netblast** in the future  **NetBLAST** searches for sequences similar to a query sequence. The query and the database searched can be either peptide or nucleic acid in any combination.  **NetBLAST** can search only databases maintained at the National Center for Biotechnology Information (NCBI) in Bethesda, Maryland, USA.  note: this is an alias for blastcl3 |
| Homepage | http://www.ncbi.nlm.nih.gov/BLAST/ |
| Remote Documentation | http://www.ncbi.nlm.nih.gov/Education/BLASTinfo/information3.html |
